# Dual-channel graph learning reveals similarity and complementarity in protein-protein interaction networks

**DOI:** 10.1371/journal.pcbi.1013941

**Published:** 2026-05-20

**Authors:** Tao Tang, Taiguang Shen, Weizhuo Li, Yangyang Chen, Sisi Yuan, Yuansheng Liu, Xinyu Yang, Xiao Luo

**Affiliations:** 1 School of Modern Posts, Nanjing University of Posts and Telecommunications, Jiangsu, China; 2 Department of Computer Science, University of Tsukuba, Ibaraki, Japan; 3 School of Chinese Medicine, Hong Kong Baptist University, Kowloon, Hong Kong SAR, China; 4 College of Computer Science and Electronic Engineering, Hunan University, Hunan, China; 5 Yuelushan Laboratory, Changsha, China; 6 College of Biology, Hunan University, Hunan, China; Al-Ahliyya Amman University, JORDAN

## Abstract

Protein-protein interactions (PPIs) are governed by two fundamental interfacial mechanisms: similarity-driven, often involving symmetric structural motifs, and complementarity-driven, arising from geometric and physicochemical matching between binding surfaces. Despite their biological significance, computational models have largely overlooked the coexistence and interplay of these twofold interaction modes. Here, we introduce DMG-PPI, a dual-channel graph neural network framework that jointly models similarity and complementarity in PPI networks, extending prior heterophilous GNN concepts to explicitly disentangle these dual interaction modes. The core of the model consists of two parallel processing pathways: Alignment Message Passing (AMP), which aggregates information from proteins with similar features to capture interactions driven by shared structural patterns, and Divergence Message Passing (DMP), which emphasizes differences between proteins and identifies complementary features that may indicate physicochemical compatibility. The signals captured by AMP and DMP are integrated via an adaptive fusion strategy within each block, and the outputs of blocks are aggregated using the MixHop framework to encode higher-order interaction patterns. DMG-PPI substantially outperforms state-of-the-art methods on classical benchmark datasets, achieving a 7.19% improvement in Micro-F1 over the second-best method. Additionally, the dual-channel framework provides interpretable insights into key binding residues by identifying interfacial mechanisms. Overall, DMG-PPI serves as a powerful tool that reveals the mechanisms behind accurate PPI predictions and facilitates downstream biological analysis.

## Author summary

Proteins interact through multiple interfacial principles rather than a single uniform mechanism, reflecting diverse modes of molecular recognition between binding partners. Accurately modeling this heterogeneity remains a central challenge in protein–protein interaction (PPI) prediction. Existing computational approaches often overlook such complexity and treat all interactions as arising from a dominant pattern. To address this limitation, we present DMG-PPI, a dual-channel graph neural network framework designed to account for distinct interaction principles within a unified model. By modeling both similarity-based and complementarity-based interaction signals, DMG-PPI enables a more comprehensive representation of interaction patterns in PPI networks. Evaluations on widely used benchmark datasets demonstrate that our approach achieves robust and consistent improvements over existing methods. Beyond prediction accuracy, DMG-PPI provides biologically meaningful interpretability by highlighting key residues and revealing distinct interfacial interaction mechanisms, offering valuable insights for downstream structural and functional studies.

## Introduction

Protein–protein interactions (PPIs) constitute the structural and functional backbone of cellular systems, coordinating molecular processes ranging from signal transduction to metabolic regulation [1,2]. PPI networks thus offer a systems-level view of how molecular interactions give rise to cellular function and how their perturbation contributes to disease pathogenesis. Mapping and modeling these networks is central to both fundamental biology and biomedical research, enabling insights into molecular organization [3], complex assembly [4], system-level cellular behavior [5], target prioritization [6], and therapeutic intervention [7]. While experimental methods have produced substantial PPI data, their expense, contextual variability, and scalability constraints underscore the need for computational approaches that can predict interactions across entire networks.

At a fundamental level, protein binding is governed by two non-mutually exclusive interaction modes. **Similarity-driven interactions** arise from shared evolutionary or biochemical features that favor compatible binding affinities [8,9], whereas **complementarity-driven interactions** depend on precise geometric and physicochemical matching at binding interfaces [10,11]. Experimental evidence supports the coexistence of these interaction modes: Yeast two-hybrid assays preferentially detect interactions between proteins with similar domain architectures [12,13], whereas X-ray crystallography reveals complementary binding at atomic resolution [14]. This duality is not unique to PPIs but recurs across biological networks, including gene regulatory [15–17], signaling [18–20], and metabolic systems [21,22], highlighting a general organizing principle of biological systems. This simplification reflects a deeper conceptual mismatch between biological interaction mechanisms and their computational abstraction. In particular, PPI networks cannot be faithfully represented by models that conflate similarity and complementarity into a single interaction logic. Consequently, the dual nature of PPI formation has not been systematically formulated as co-equal, first-class modeling principles in computational interactomics, limiting our ability to disentangle, interpret, and reason about distinct interaction modes at the network level.

Currently, the research in computational PPI prediction primarily addresses two key aspects: learning informative representations of individual protein from their sequences or structures [23, 24], and modeling the interaction patterns within PPI networks [25]. The former aspect primarily relies on physicochemical characteristics [26,27] and residue-level encoding [28,29] of protein sequences to generate protein embeddings. More recently, Gao et al. [30] introduced graph neural networks (GNNs) to model protein tertiary structures as graphs. Graph autoencoder and masked modeling was then applied to learn a robust representation of protein structure [31]. The latter aspect concentrates on modeling interactome using GNN. Graph Isomorphism Network (GIN) was among the first GNN architectures applied to PPI prediction [32]. Subsequent studies have demonstrated improvements in generalization capabilities through Graph Attention Network (GAT) [33] and anti-symmetric Graph Convolutional Network (GCN) [34], as well as incorporating GNNs into broader frameworks [35,36].

While graph neural networks (GNNs) have become widely used for PPI prediction, most existing architectures primarily aggregate features from similar neighbors. This design inherently favors the detection of similarity-driven interactions and makes it difficult for them to recognize interactions based on complementarity. Although prior work on heterophilous GNNs has introduced mechanisms to handle structurally or functionally dissimilar nodes, these approaches were not specifically designed to disentangle the co-occurrence of similarity- and complementarity-driven interactions inherent to PPI networks. This gap constrains both predictive accuracy and biological interpretability.

Here, we introduce DMG-PPI, a Dual Message Pathway Graph Neural Network that explicitly models the dual principles governing protein–protein interactions. DMG-PPI decomposes message passing into two parallel channels: Alignment Message Passing (AMP), which captures similarity-driven interactions, and Divergence Message Passing (DMP), which captures complementarity-driven interactions through contrasting features. These channels are processed in parallel and adaptively fused to generate multi-scale representations that preserve the distinct biological semantics of each interaction mode. The AMP channel follows the homophily assumption by aggregating features from similar neighbors through summation, while the DMP channel extends heterophilous GNN principles through multiplicative message passing that explicitly contrasts node features, enabling the capture of complementary binding patterns that are biologically distinct from simple feature dissimilarity. By grounding network modeling in the physical principles of protein binding, DMG-PPI achieves superior predictive accuracy, robustness to noise, and enhanced interpretability, enabling inference of dominant binding modes and key interface features within PPI networks.

Beyond the methodological advances introduced in this work, the underlying biological question—how proteins recognize and bind to their partners—is fundamental to molecular biology and of broad interest to experimental researchers. Protein-protein interactions govern nearly all cellular processes, from signal transduction to immune responses, yet the principles that determine why certain proteins interact while others do not remain incompletely understood. By explicitly modeling two distinct interaction mechanisms—similarity-driven binding, where proteins with shared structural features associate, and complementarity-driven binding, where proteins with matching but different features interact—our framework aims to capture biological reality more faithfully than previous approaches. For experimental biologists, DMG-PPI offers practical utility beyond prediction accuracy: the model can highlight which residues are likely critical for binding (hotspots) and indicate whether a given interaction likely arises from structural similarity or physicochemical complementarity, providing testable hypotheses for mutagenesis or structural studies.

## Materials and methods

**Fig 1:**
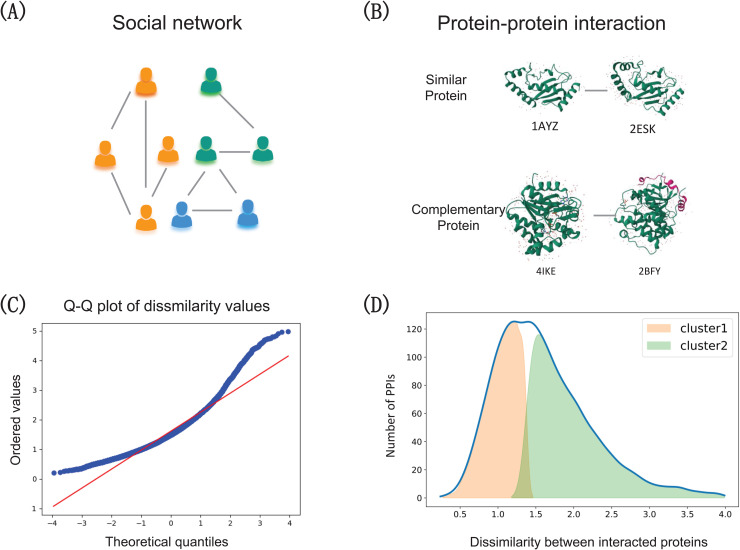
Protein–protein interactions (PPIs) can emerge through two principal mechanisms: similarity and complementarity. **(A)** In social networks, individuals with similar attributes tend to connect, a phenomenon termed homophily. **(B)** Analogously, in PPI network, both similar and complementary proteins tend to interact. **(C)** A Q-Q plot, a statistical tool for comparing distributions, confirms this pattern. The fact that many points deviate from the straight reference line indicates that the interacting protein pairs do not all follow a single, simple rule, but instead represent a mix of different interaction types. **(D)** Clustering analysis further categorizes the binding protein pairs into two groups exhibiting markedly different levels of structural dissimilarity. These groups correspond to interactions primarily driven by structural similarity and complementarity, respectively.

Homophily is a fundamental assumption in graph theory, referring to the tendency of links to form between similar nodes. This property is prevalent in many studies on graph structure, such as social network (Fig 1A). Most previous studies of PPI networks have largely relied on this assumption, favoring GNN architectures with sum-based aggregation that naturally emphasize homophilous relationships. However, as illustrated in Fig 1B, biological evidence indicates that protein interactions often arise not only between structurally or functionally similar proteins but also between complementary counterparts, reflecting the coexistence of similarity- and complementarity-driven interactions.

To systematically investigate protein interaction patterns, we quantified the similarity between interacting proteins as the euclidean distance between their embeddings derived from pre-trained geometric model [31]. A Q–Q plot of similarity scores for interacting pairs in SHS27K is shown in Fig 1C, where numerous points deviate substantially from the reference line, suggesting a mixed distribution of similarity among interacting proteins. Subsequent clustering analysis (Fig 1D) further supports the coexistence of multiple connection patterns in PPI networks. Specifically, we computed pairwise similarity scores for interacting protein pairs and examined their empirical distribution. The resulting distribution exhibits two distinct peaks, forming a clear bimodal pattern. This bimodality suggests that the interaction samples naturally partition into two separable groups: one characterized by high similarity, consistent with similarity-driven interactions, and the other characterized by lower similarity, indicative of complementarity-driven relationships. The presence of two stable density modes indicates that protein interactions cannot be explained by a single uniform mechanism, but instead arise from at least two major binning principles. Together, these findings highlight the complex nature of PPIs, in which similarity-based and complementary mechanisms jointly contribute to network connectivity.

**Fig 2:**
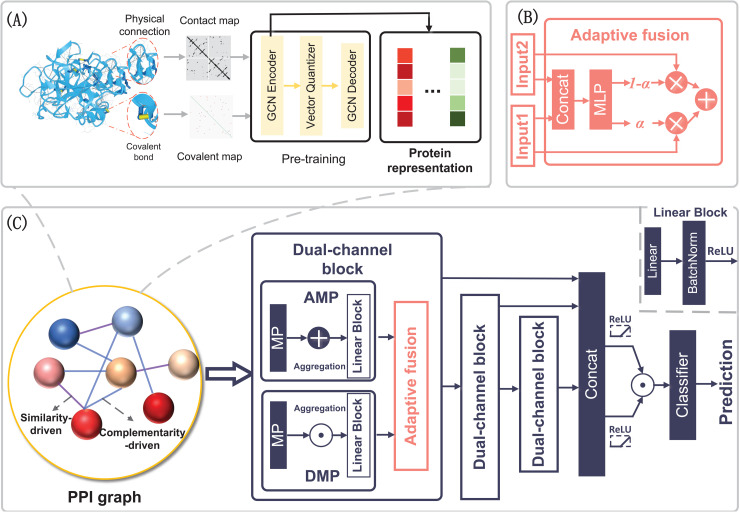
(A) Each protein is first converted into a structural representation derived from its contact map and covalent connectivity. These structural features are encoded into compact protein embeddings using a pre-trained graph neural network autoencoder. In simple terms, this step transforms complex structural information into a learnable numerical representation of each protein. **(B)** The adaptive fusion block integrates different sources of structural information using a gating mechanism, which automatically determines how much weight to assign to each feature type depending on the context. **(C)** The core of DMG-PPI models two complementary principles of protein interactions. AMP captures similarity-based relationships, where proteins with similar structural patterns tend to interact. DMP captures complementarity-based relationships, where structurally distinct but functionally compatible proteins interact. The outputs of these channels are combined to form refined protein representations. Finally, the interaction between two proteins is quantified by combining their representations into a pair-level descriptor, which is used to predict whether the proteins interact.

Here, we propose DMG-PPI for modeling the two-fold interaction patterns in PPI network. As depicted in Fig 2, our approach comprises four main modules: (1) construction of the physical protein graph, (2) a graph autoencoder for data preprocessing and generation of initial node embeddings, (3) a dual-channel GNN framework for capturing complex interaction patterns within the PPI network, and (4) a classifier for final prediction.

### Problem formulation

The PPI network is denoted as 𝒢=(P,I), where $𝒫=\{p_{1},p_{2},\ldots ,p_{n}\}$ is the protein set, and $ℐ=\left(p_{i},p_{j},y_{ij}\right)$, the protein feature of *p*_*i*_ is defined as *x*_*i*_. The objective is to learn a model $(p_{i},p_{j})\to \hat{y_{ij}}$ from training set.

### Feature extraction from intramolecular physical and chemical connections

We use residue-level graph to model the tertiary structure of each protein. Unlike the previous methods that only learn the spatial contact inside protein, our approach considers both the physical and chemical connections. For each protein, two residual-level protein graphs are constructed to model the physical and chemical connections respectively, both graphs consist of an adjacency matrix $A_{p}\in \{0,1{\}}^{r\times r}$ and a feature matrix $M_{p}\in {\mathbb{R}}^{r\times s}$, where *r* denotes the residue number of protein.

For chemical protein graph, the adjacency matrix is derived from covalent bonds between atoms; if there is a covalent bond between two atoms, then there is a connection between the corresponding residues. Covalent bonds are identified based on known chemical bonding patterns within amino acid residues and ligand molecules. For each pair of atoms, the connection is determined by the presence of a covalent bond as defined by standard residue chemistry or explicit bonding information in the structure data. The node feature is formed by three physicochemical properties: solvent accessible surface area (SASA), electrostatic potential surface (EPS), and pKa shifts. For the physical connection protein graph, a contact map is used as the adjacency matrix, in which the connection between residues is determined by the 3D coordinates of alpha carbon atom in residue. For each pair of residue, the connection exists if the distance between their alpha carbon atoms is smaller than 8Å. The node features are extracted by the type-dependent encoding of RDKit [37].

All computed node features are subsequently normalized across the dataset prior to model input to ensure numerical stability and comparability across proteins. The physical and chemical protein graphs are processed with graph autoencoder to learn the embeddings of protein graph, we adapt the masked vector quantizer [31] for training process. The protein graph are processed by GNN encoder, GNN decoder and a vector quantizer:


$$\begin{array}{cc} & Z={GNN}_{\text{enc}}(M_{p},A_{p})\\ & Q=VQ(Z)\\ & \hat{M_{\text{p}}}={GNN}_{\text{dec}}(Z,Q), \end{array}$$ (1)

the masked modeling *m* is applied on output of vector quantizer:


$$\begin{array}{cc} & Q_{\text{mask}}=m⊙Q,m\in \{0,1{\}}^{d}\\ & {\hat{M}}_{\text{recon}}={GNN}_{\text{dec}}(Z,Q_{\text{mask}}). \end{array}$$ (2)

The graph autoencoder is pre-trained on SHS148K. The reconstruction loss is defined as the sum of three components: the mean squared error (MSE) between quantized representations *Q* and latent representation *Z*; the MSE between the original feature *M*_*p*_ and its reconstruction $\hat{M_{\text{p}}}$; and the MSE between original feature *M*_*p*_ and masked reconstruction ${\hat{M}}_{\text{recon}}$.

The embedding of each protein *h*_*i*_ is obtained by concatenating the latent representations from the physical and chemical graphs, followed by a readout operation: $h_{i}=\text{READOUT}(Z_{\text{phy}}\|Z_{\text{che}})$, ‖ denotes the concatenation operation.

### Dual-channel framework for learning interaction pattern

DMG-PPI employs two parallel channels to jointly model similarity- and complementarity-driven interactions simultaneously. Given a PPI graph 𝒢=(𝒫,ℐ), along with the initial node embeddings **h** for each protein, DMG-PPI propagates information through a stack of dual-channel GNN blocks, ${𝐡}_{i}^{l+1}$ denotes the output of *l*th block. Within each block, protein representations are independently processed through two specialized message-passing pathways: AMP, designed to capture similarity-driven interactions, and DMP, tai*l*ored for complementarity-driven interactions. The outputs of AMP and DMP are formally defined as:


$$\begin{array}{cc} & {𝐡}_{i,\text{AMP}}^{\left(l+1\right)}=\sigma \left(\sum_{j\in 𝒩(i)\cup \{i\}}\frac{1}{\sqrt{d_{i}d_{j}}}{𝐖}_{\text{AMP}}^{\left(l\right)}{𝐡}_{j}^{\left(l\right)}\right)\\ & {𝐡}_{i,\text{DMP}}^{\left(l+1\right)}=\sigma \left(\underset{j\in 𝒩(i)\cup \{i\}}{⨀}{𝐖}_{\text{DMP}}^{\left(l\right)}{𝐡}_{j}^{\left(l\right)}\right), \end{array}$$ (3)

where ${𝐖}_{\text{AMP}}^{\left(l\right)}$ and ${𝐖}_{\text{DMP}}^{\left(l\right)}$ denote the learnable weight matrix of each channel, 𝒩(i) denotes the neighborhoods of *p*_*i*_, *d*_*i*_ denotes the degree of the *i*th protein. AMP and DMP employ additive and multiplicative message-passing pathways, respectively, to capture the distinct biological characteristics of PPI networks. AMP aggregates features from neighboring proteins through summation, which smooths the representations and naturally reinforces similarity-driven interactions. In contrast, DMP emphasizes physicochemical complementarity between interacting proteins. By combining neighbor features multiplicatively, DMP amplifies contributions from proteins with complementary structural or functional characteristics, reflecting the underlying biophysical principle that complementary surfaces or motifs facilitate stable interactions.

A gating mechanism is introduced to adaptively balance the contribution of both channels. The gate coefficient α∈(0,1) is obtained by linearly projecting the concatenated AMP and DMP embeddings:


$$\begin{array}{c} \alpha =\sigma \;\left(W_{g}\cdot [{𝐡}_{i,\text{AMP}}^{\left(l+1\right)}\;\|\;{𝐡}_{i,\text{DMP}}^{\left(l+1\right)}]+b_{g}\right), \end{array}$$ (4)

where *W*_*g*_ and *b*_*g*_ are learnable parameters, σ denotes an activation function that constrains α in range (0,1). In this study, we employ sigmoid function. This coefficient adaptively regulates the relative contribution of each channel to the fused representation, as follows:


$$\begin{array}{c} {𝐡}_{i}^{\left(l+1\right)}=\sigma (BN(\alpha \cdot {𝐡}_{i,\text{AMP}}^{\left(l+1\right)}+(1-\alpha )\cdot {𝐡}_{i,\text{DMP}}^{\left(l+1\right)})). \end{array}$$ (5)

To capture multi-scale dependencies and alleviate the over-smoothing effects commonly observed in deep GNNs, we adopt a MixHop-based aggregation scheme for computing the final protein representation. In the MixHop framework, the embedding of each block is concatenated together, followed by linear transformation, batch normalization and activation function sequentially. The final node embedding is as following:


$$\begin{array}{c} x_{i}=\sigma (\text{BN}(W_{c}\cdot [h_{i}^{1}\|h_{i}^{2}\|...\|h_{i}^{l}]+b_{c})). \end{array}$$ (6)

### Classifier and training

For prediction, the representation of candidate protein pair (*p*_*i*_, *p*_*j*_) is derived by element-wise multiplication of their embeddings, followed by a three-layer multilayer perceptron (MLP):


$$\begin{array}{c} {\hat{y}}_{ij}=\text{MLP}(x_{i}⊙x_{j}). \end{array}$$ (7)

The task is formulated as a multi-label classification problem, in which each pair of proteins may participate in multiple types of PPIs. We utilize a multi-task binary cross-entropy loss for training, defined as:


$$\begin{array}{c} ℒ=\sum_{x_{ij}\in {𝒳}_{\text{train}}}\frac{1}{C}\sum_{k=1}^{C}\ell_{\text{BCE}}(y_{ij}^{k},{\hat{y}}_{ij}^{k}), \end{array}$$ (8)

where *C* is the number of PPI types, $y_{ij}^{k}\in \{0,1\}$ denotes the ground-truth label for type *k*, ${\hat{y}}_{ij}^{k}$ is the predicted probability, and $\ell_{\text{BCE}}(y,\hat{y})=-y\log\hat{y}-(1-y)\log(1-\hat{y})$.

## Results

We benchmark DMG-PPI on SHS27K and SHS148K [30,33,38,39], two canonical multi-type PPI datasets curated from STRING [40], a comprehensive resource for human interactome analysis. Both datasets were filtered to retain only high-confidence interactions (score ≥ 0.7), resulting in 12,517 PPIs in SHS27K and 44,488 PPIs in SHS148K. To minimize protein overlap between the training and test sets, we adopt the Breadth-First Search (BFS) and Depth-First Search (DFS) splitting strategies proposed by Lv et al. [39]. During the experiments, DMG-PPI was trained with a learning rate of 1 × 10^−3^ and a weight decay of 5 × 10^−4^. The number of layers was set to three, and the hidden dimension was fixed at 256, without applying dropout. Training was conducted using mini-batch gradient descent, and early stopping was employed based on the validation loss. Specifically, training was terminated if no improvement was observed for 20 consecutive epochs.

The benchmark methods include PIPR [41] (Siamese residual recurrent convolutional network), ESM2_AMP [42] (Uses the ESM2 protein language model to extract sequence segment feature), TUnA [43] (Transformer-based Uncertainty Aware model for PPI), GNN_PPI [32] (1d convolution and graph convolution), AFTGAN [33] (attention‑free transformer and graph attention mechanisms), LDMGNN [44] (Graph neural network‑based method for multi‑graph learning of PPIs), HIGH-PPI [30] (Hierarchical PPI prediction model that leverages multi‑level graph information to capture interaction topology), BaPPI [34] (Anti‑symmetric GNN framework for balanced learning), and MAPE-PPI [31] (Microenvironment‑aware protein embedding model that encodes residue environments with structural context).

### DMG-PPI achieves the best performance and model capabilities

**Fig 3:**
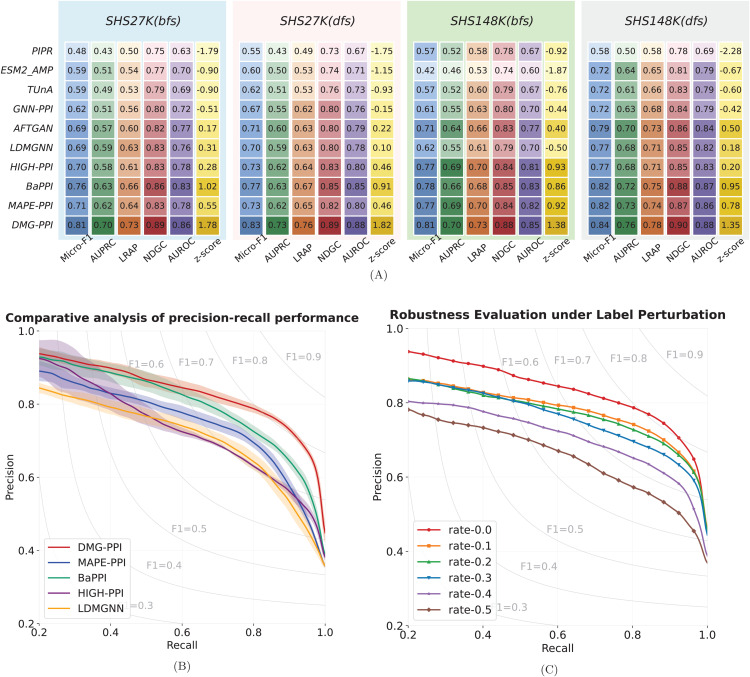
Comparative performance of DMG-PPI and benchmark methods. **(A)** Heatmaps showing the performance of each method across the following evaluation metrics: Micro-F1, AUPRC, LRAP, NDCG, AUROC, and overall z-score. Darker colors indicate better performance. Each row indicates the performance of corresponding method. **(B)** Precision–recall curves for PPI prediction on SHS27K, comparing the top five methods; shaded areas represent the range between the highest and lowest results. **(C)** Robustness of DMG-PPI to label perturbation on SHS27K, evaluated using precision–recall curves at different perturbation ratios.

Fig 3A presents the results of DMG-PPI compared with leading benchmark methods. Each experiment was repeated five times, with mean values (two decimal places) reported; standard deviations are provided in S1–S4 Tables. Prediction performance is quantified by: (i) Micro-F1: the harmonic mean of precision and recall; (ii) AUPRC: area under the precision–recall curve; (iii) NDCG: normalized discounted cumulative gain; (iv) P@500L: proportion of positive PPIs among the top 500 ranked links; (v) AUROC: area under the receiver operating characteristic curve; and (vi) z-score aggregated across the above metrics.

DMG-PPI consistently outperforms all state-of-the-art methods across every metric. MAPE-PPI and BaPPI achieve competitive second-place performance in different scenarios, while PIPR demonstrates notably inferior performance, likely due to its non-GNN architecture, which limits its ability to capture global topological information. Given the high class imbalance and skewed link distribution in PPI datasets, Micro-F1 provides a more reliable performance measure than NDCG or AUROC, as the latter tend to overestimate performance when generalization is weak [45]. This explains the smaller differences observed between methods under NDCG and AUROC. Quantitatively, DMG-PPI exceeds the second-best method in Micro-F1 by 7.19% on SHS27K and 3.14% on SHS148K.

We further evaluate robustness by introducing label perturbations into the training set to simulate undiscovered PPIs, which is a common feature of PPI datasets. Positive labels for each interaction were randomly removed at ratios ranging from 10–50% to simulate the frequently occurring undiscovered interactions. As shown in Fig 3C, DMG-PPI sustains an F1-score above 0.7 even with 30% label removal, with negligible performance loss when the ratio is below 30%. This resilience indicates that DMG-PPI captures stable global interaction patterns that persist despite incomplete supervision, particularly in high-recall regimes. At low recall, the model trained with complete labels retains a clearer advantage, underscoring robustness in top-ranked predictions.

**Fig 4:**
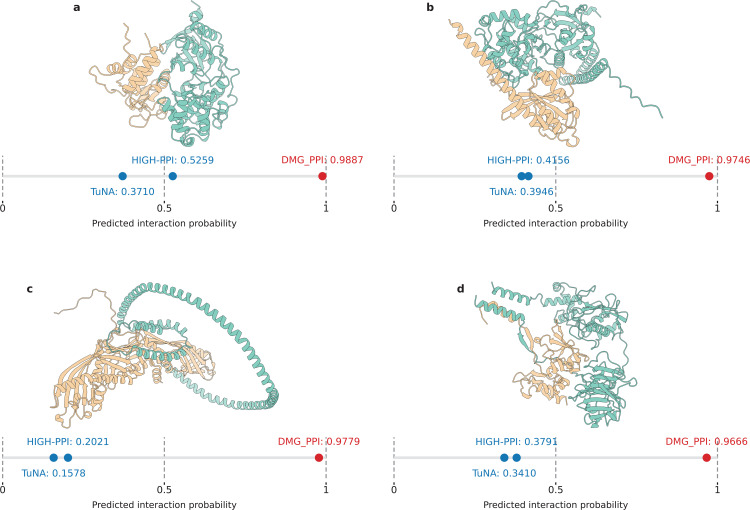
Protein–protein interaction (PPI) examples that are correctly predicted by DMG-PPI but not by HIGH-PPI or TUnA. The corresponding PPI complex structures are predicted using GRAMM and visualized with ChimeraX. **(A)** O15264 (Mitogen-activated protein kinase 13) and P51452 (Dual specificity protein phosphatase 3); **(B)** P11233 (Ras-related protein Ral-A) and Q15208 (Serine/threonine-protein kinase 38); **(C)** P11597 (Cholesteryl ester transfer protein) and P02647 (Apolipoprotein A-I); **(D)** P01033 (Metalloproteinase inhibitor 1) and Q9H306 (Matrix metalloproteinase-27).

Notably, the superior performance of DMG-PPI can be attributed to its enhanced capability to recognize positive PPIs from the minority class. To illustrate this discrepancy, we examine four representative positive PPI cases formed by biologically important proteins. As shown in Fig 4, DMG-PPI assigns systematically higher confidence scores to true positive interactions, with the minimum predicted probability exceeding 0.96. In contrast, the predicted probabilities produced by TUnA and HIGH-PPI for the same positive PPIs fall within a much lower range of 0.20 to 0.53, with most values concentrated between 0.3 and 0.4. DMG-PPI exhibits a clear separation between positive and negative predictions, this pronounced separation in predicted probabilities highlights its superior discriminative power and reliability in identifying minority positive PPIs.

### DMG-PPI precisely detects key interface residues underlying PPIs

Hotspots are specific subsets of residues that contribute disproportionately to the binding free energy, hence play a fundamental role in governing protein–protein interactions. The divergence message pathway (DMP) focuses on physicochemical and geometric complementarity at the protein interface, which are key factors underlying the formation of hotspots. The residue-level DMP scores allow estimation of the probability that a residue serves as such a site.

**Fig 5:**
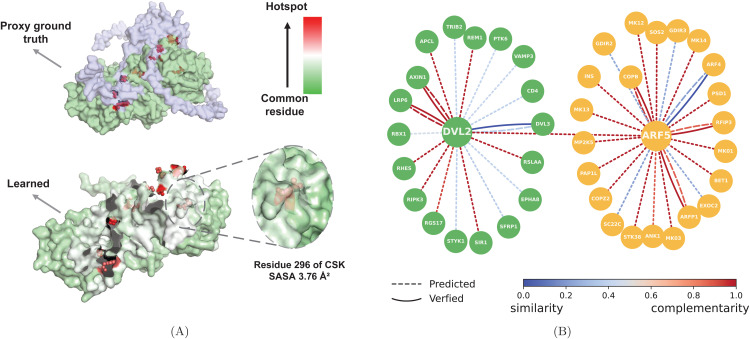
The interpretability provided by DMG-PPI. **(A)** Surface representation of a protein complex (UniProt IDs: O76528 and P41240). The likelihood of a residue being a hotspot is visualized using a color gradient from low (green) to high (red). **(B)** DMG-PPI accurately identifies the interaction mode between proteins. Dashed lines represent the prediction computed by DMG-PPI, while solid lines represent ground-truth. The modes of PPIs are represented by edge color, varying along a spectrum from blue (similarity-driven) to red (complementarity-driven).

We systematically mask each residue individually and compute the resulting percentage change in both DMP and the prediction. A normalized Bayesian-style product of these two changes is then used to quantify the likelihood of each residue being a hotspot. We provide a case study of hotspot probability of interaction between HMBOX1 (UniProt ID O76528) and CSK (UniProt ID P41240). The proxy ground truth is computed by Rosetta Flex ddG. As shown in Fig 5A, the tendency of being hotspot is represented by color, DMG-PPI identifies nearly all hotspots with high accuracy. In particular, it successfully detects hotspots with low SASA (e.g., residue 296 of CSK, 3.76 Å²), which exhibit limited structural exposure and are often overlooked by conventional methods that rely solely on surface geometry. The ability of DMG-PPI to recognize these less apparent, structurally buried hotspots underscores its broader applicability and robustness in protein–protein interaction analysis.

Furthermore, since the two channels of DMG-PPI correspond to shape complementarity and structural motifs, respectively, their associated gating coefficients can be leveraged to infer the dominant driving force of PPI. We provide a case study of the driving force that relates two proteins: DVL2, a well-connected, high-centrality node in Wnt signaling networks [46,47], and ARF5, an essential functional protein [48] involved in vesicular trafficking, the ground truth is obtained from IntAct [49]. The confidence score of driving force is derived from the gating coefficients in our dual-channel framework. As shown in Fig 5B, experimentally verified driving force is represented by solid line, while prediction is indicated by dashed line, the color of edges demonstrate the confidence score of driving force. DMG-PPI correctly predicts the main driving force of all PPIs with available ground truth, with particularly strong accuracy observed for interactions such as DVL2–LPR6, DVL2–AXIN1, and ARF5–COPB. The accurate prediction provided by DMG-PPI can relieve the high cost of experimental method, the excellent interpretability also demonstrates DMG-PPI learns generalized protein representation.

### Learning dual interaction patterns enhances performance

The key innovation of our approach lies in a dual-channel framework that integrates distinct message-passing pathways, each tailored to a specific interaction pattern in the PPI network. To assess the contribution of learning dual interaction patterns, we compare the performance of the full dual-channel model with its single-channel variants, where only the AMP or DMP is used. The evaluation is conducted from the following perspectives: (1) an ablation study examining the individual contribution of each channel, (2) a type-specific performance analysis in terms of AUPR, and (3) an analysis of the interaction patterns among the top 500 predicted PPIs.

**Fig 6:**
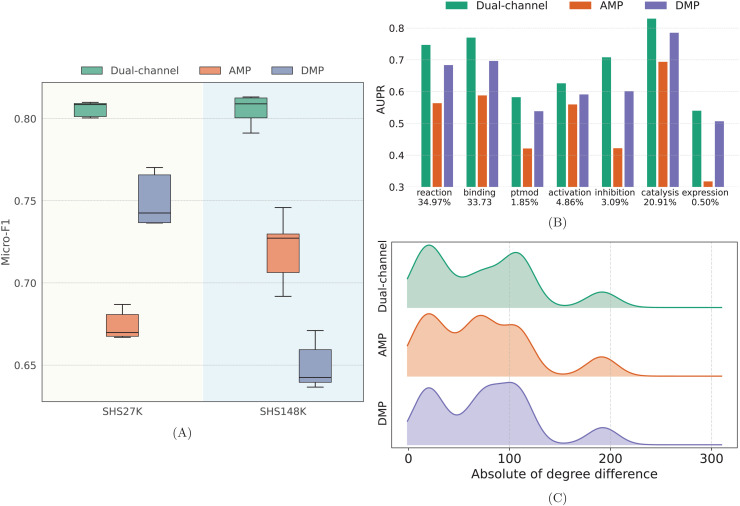
Performance comparison between each message pathway. **(A)** The Micro-F1 of each variant on SHS27K and SHS148K. **(B)** The AUPR across PPI types on SHS27K, the x-axis labels denote PPI types and the corresponding percentage. **(C)** Patterns of top-500 PPIs predicted by each variant. The x-axis represents the absolute value of the degree difference between the interacting proteins, while the y-axis indicates the percentage.

We first examine the performance of each channel on SHS27K and SHS148K. As illustrated in Fig 6A, the dual-channel framework substantially outperforms each individual message pathway on both datasets. These results indicate that each message pathway is limited to learning only a portion of the structural information inherent in the PPI network, thereby verifying the coexistence of interaction patterns and the effectiveness of our approach. Moreover, the variance of dual-channel framework is considerably lower than that of AMP and DMP, highlighting the model’s ability to achieve balanced learning across the two interaction patterns and its strong robustness. Notably, AMP performs better on SHS148K, whereas DMP yields more accurate predictions on SHS27K. This discrepancy may be attributed to the higher similarity among interacting proteins in SHS148K compared to SHS27K, which makes similarity-driven interactions more prominent in the former.

We further evaluate each channel’s performance across interaction types on the SHS27K dataset. In PPI networks, the distribution of interaction types is highly imbalanced: the dominant types (reaction, binding, and catalysis) account for over 80% of all interactions. Such skewed distribution may bias prediction models, making balanced performance across types a meaningful indicator of generalization. As shown in Fig 6B, the dual-channel framework significantly outperforms AMP and DMP across all interaction types. The performance gains of the dual-channel over AMP are especially pronounced for binding, ptmod, inhibition, and expression types that are strongly associated with biochemical complementarity (e.g., charge, shape, and hydrophobicity). These results demonstrate that our approach effectively addresses the gap in learning complementarity patterns within PPI networks. Particularly, ptmod interactions hold considerable biological significance, as they are central to protein function regulation, signal transduction pathways, and disease mechanisms. Improvements in predicting these interactions may thus have broad implications for downstream biomedical applications, including disease gene prioritization and drug target identification.

To examine whether the learned gating coefficient reflects similarity-driven interaction patterns, we computed the Spearman correlation between α and sequence identity for binding interactions in SHS148K. The analysis yielded a moderate positive correlation (ρ = 0.377, *p* = 0.071), indicating a trend in which higher sequence similarity corresponds to increased weighting of the similarity-driven (AMP) pathway. Although not statistically significant at the 0.05 level, this result supports the interpretation of α as capturing interaction mode preference

Finally, to probe the interaction patterns captured by each pathway, we analyze the top 500 predicted PPIs from the dual-channel, AMP, and DMP models on SHS27K. Specifically, we compare the absolute degree difference between interacting protein pairs. As illustrated in Fig 6C, certain recurring patterns emerge, such as peaks around degrees 25, 100, and 175. The distribution associated with the dual-channel model is more densely concentrated than those of AMP and DMP, suggesting that it captures more distinct structural characteristics and integrates them more effectively.

### DMG-PPI reveals the relationship between PPI types

In applications such as mechanistic modeling and pathway reconstruction [50], information about individual PPI types is often insufficient. Investigating the relationships between different PPI types can yield deeper mechanistic insights into molecular functions and disease mechanisms. To explore these inter-type functional dependencies, we decompose the classifier’s weight matrix into a set of *k* × *k* vectors based on the corresponding PPI type. The L2 norm of each vector is used to quantify the influence between PPI types.

**Fig 7:**
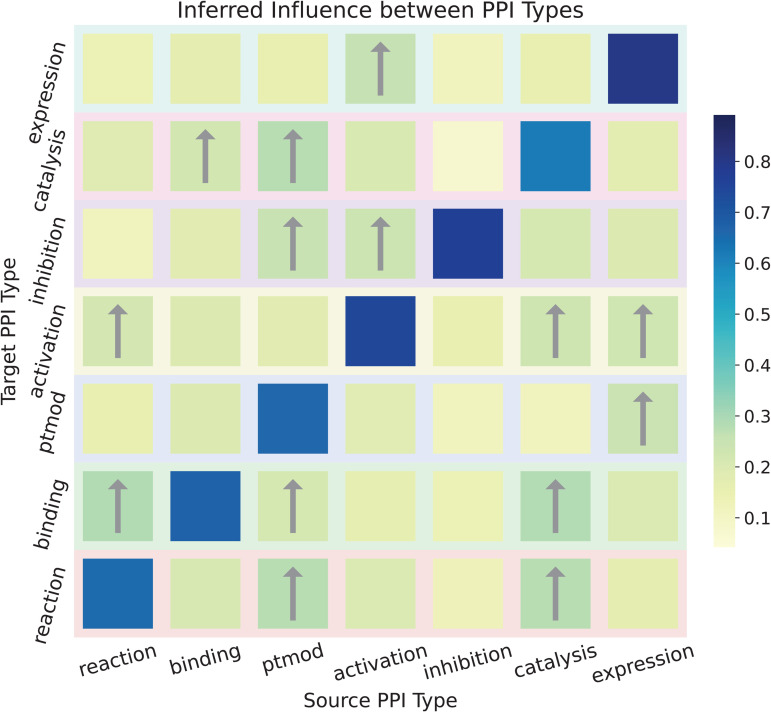
Directional influence among PPI types. Each row corresponds to a specific PPI type, while each column represents the type influencing it. The value at row *i* and column *j* indicates how strongly type *j* contributes to type *i*, as learned by DMG‑PPI. Arrows mark influences stronger than 0.2. Overall, the map highlights directional relationships among interaction types and reflects known biological mechanisms.

The inferred influence map produced by DMG-PPI is presented in Fig 7, where each row indicates how all PPI types contribute to a specific PPI type. Notably, the inferred relationships align with some known biological mechanisms. For example, the reaction type is most influenced by ptmod and catalysis, consistent with previous findings that reaction events frequently occur in catalytic or modifying contexts [51,52]. The matrix is notably asymmetric, revealing directional dependencies rather than reciprocal relationships. For instance, ptmod strongly influences reaction, whereas the reverse effect is comparatively weaker, consistent with their mechanistic hierarchy. Quantitatively, reaction, binding, ptmod, and catalysis exhibit the highest aggregated incoming influence scores, indicating that these types are more context-dependent.

### DMG-PPI learns topology-robust representations beyond local motif density

**Fig 8:**
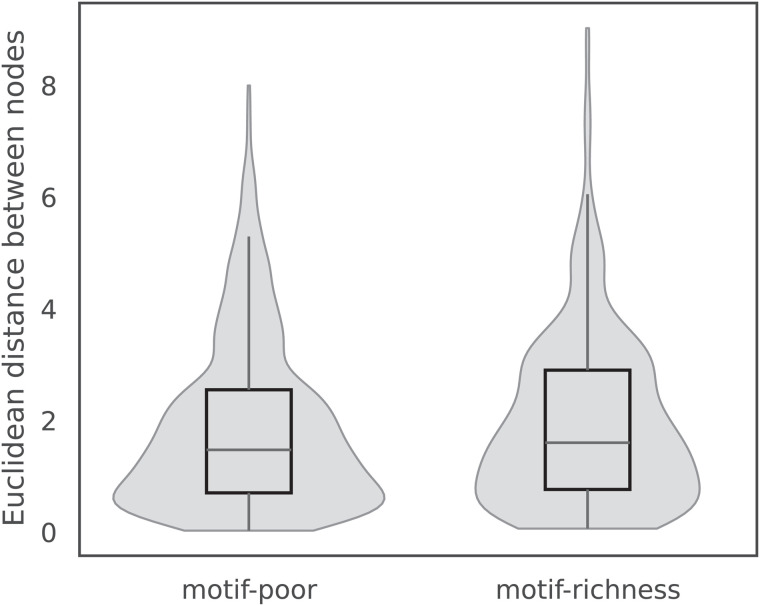
Distribution of Euclidean distances between node embeddings for edges in motif-richness versus motif-poor regions. The similarity of distances across regions suggests that DMG-PPI learns representations largely independent of local motif density.

To assess whether DMG-PPI learns unbiased representations that are not strongly influenced by local network topology, we compared embedding distance distributions for edges located in motif-dense versus motif-poor neighborhoods. The motif density of each edge was quantified as the sum of L3 motifs associated with its two endpoints. Edges in the bottom 20% and top 20% of motif density were defined as motif-poor and motif-richness, respectively. As shown in Fig 8, the resulting embedding distance distributions largely overlap, with only minor differences observed between the two groups. These findings suggest that DMG-PPI does not preferentially encode interactions in dense topological motifs, but instead learns representations that are relatively insensitive to local motif overrepresentation, consistent with a balanced integration of network context and protein-specific features.

Further, linear regression analysis indicates that motif richness explains only 1.6% of the variance in embedding distances (*R*^2^ = 0.016), suggesting that DMG-PPI embeddings are largely independent of local motif density. Consistently, Spearman correlation analysis reveals a weak monotonic relationship between motif richness and embedding distance (ρ=0.10, *p* = 1.72 × 10^−8^), highlighting that the relative organization of edges in the embedding space is minimally influenced by motif structure. Together, these results demonstrate that DMG-PPI captures topology-robust representations that extend beyond the influence of local motif density.

## Discussion

In this paper, we introduce DMG-PPI, an efficient PPI prediction method that models the complex interaction patterns in PPI networks from a dual-channel GNN perspective. In the preprocessing stage, the physical connection and covalent bond are extracted as initial protein representation. At interactome level, we employ two channels that use additive and multiplicative aggregation to capture complementarity-driven and similarity-driven interactions, respectively. Experiments on classical benchmark datasets demonstrate that DMG-PPI significantly outperforms state-of-the-art methods in PPI prediction. Our framework also proves to be highly reliable, performing well even on rarer types of PPIs and when the input data is incomplete or noisy. Most importantly for biological discovery, DMG-PPI offers clear interpretability: by analyzing its internal calculations, we can identify whether an interaction is likely driven by similarity or complementarity and pinpoint the specific residues involved, providing concrete, testable hypotheses for experimental follow-up. Overall, the dual-channel framework provides a powerful tool for systematic characterization of interactome connectivity and enhances mechanistic insights into PPIs.

While DMG-PPI achieves promising performance, some areas are worth consideration for further improvements. (1) This study primarily investigated the driving forces of PPIs in the context of binary interactions. However, in multiprotein complexes, the overall stability and functionality often emerge from a mosaic of interaction modes across different protein pairs. Modeling these interdependencies could provide a more faithful representation of the biophysical principles governing complex formation. (2) The potential of a unified protein graph that simultaneously incorporates physical and chemical features remains unexplored. A geometric GNN that directly operates on the full 3D atomic coordinates—integrating shape, electrostatics, and residue-level properties into a single graph could enable the learning of physically interpretable embeddings, potentially improving generalization to unseen proteins. (3) Although DMG-PPI employs a dual-channel GNN to capture both similarity- and complementarity-driven interactions, its effectiveness still depends on the availability of informative topological signals in the interaction network. For proteins located in relatively sparse or atypical regions of the network, the limited neighborhood information may reduce the effectiveness of graph propagation, potentially leading to missed or low-confidence predictions for certain interactions. (4) In addition, current multi-label formulation treats non-annotated interaction types of known protein pairs as negative labels and does not incorporate non-interacting protein pairs. Given the incompleteness of PPI annotations, this assumption may introduce potential bias. Developing more refined negative sampling or confidence-aware labeling strategies may further enhance robustness and biological validity.

## Supporting information

S1 Table Detailed performance comparison of DMG-PPI and benchmark methods on SHS27K under the BFS scheme. (PDF)

S2 Table Detailed performance comparison of DMG-PPI and benchmark methods on SHS27K under the DFS scheme. (PDF)

S3 Table Detailed performance comparison of DMG-PPI and benchmark methods on SHS148K under the BFS scheme. (PDF)

S4 Table Detailed performance comparison of DMG-PPI and benchmark methods on SHS148K under the DFS scheme. (PDF)

S1 Fig Protein–protein interaction between P04004 and P05231 that is not correctly identified by existing methods. (PDF)
